# Building capacity to develop an African teaching platform on health workforce development: a collaborative initiative of universities from four sub Saharan countries

**DOI:** 10.1186/1478-4491-12-31

**Published:** 2014-05-30

**Authors:** Woldekidan Kifle Amde, David Sanders, Uta Lehmann

**Affiliations:** 1 School of Public Health, University of the Western Cape, Robert Sobukwe Road, Bellville 7535, South Africa

**Keywords:** African teaching platform, Institutional capacity development, Health workforce development, Partnership, Sustainability, Flexible delivery, South-South cooperation

## Abstract

**Introduction:**

Health systems in many low-income countries remain fragile, and the record of human resource planning and management in Ministries of Health very uneven. Public health training institutions face the dual challenge of building human resources capacity in ministries and health services while alleviating and improving their own capacity constraints. This paper reports on an initiative aimed at addressing this dual challenge through the development and implementation of a joint Masters in Public Health (MPH) programme with a focus on health workforce development by four academic institutions from East and Southern Africa and the building of a joint teaching platform.

**Methods:**

Data were obtained through interviews and group discussions with stakeholders, direct and participant observations, and reviews of publications and project documents. Data were analysed using thematic analysis.

**Case description:**

The institutions developed and collaboratively implemented a ‘Masters Degree programme with a focus on health workforce development’. It was geared towards strengthening the leadership capacity of Health ministries to develop expertise in health human resources (HRH) planning and management, and simultaneously build capacity of faculty in curriculum development and innovative educational practices to teach health workforce development. The initiative was configured to facilitate sharing of experience and resources.

**Discussion:**

The implementation of this initiative has been complex, straddling multiple and changing contexts, actors and agendas. Some of these are common to postgraduate programmes with working learners, while others are unique to this particular partnership, such as weak institutional capacity to champion and embed new programmes and approaches to teaching.

**Conclusions:**

The partnership, despite significant inherent challenges, has potential for providing real opportunities for building the field and community of practice, and strengthening the staff and organizational capacity of participant institutions. Key learning points of the paper are:

• the need for long-term strategies and engagement;

• the need for more investment and attention to developing the capacity of academic institutions;

• the need to invest specifically in educational/teaching expertise for innovative approaches to teaching and capacity development more broadly; and

• the importance of increasing access and support for students who are working adults in public health institutions throughout Africa.

## Background

The central place of human resources for health (HRH) in providing universal and equitable health care coverage has been acknowledged. It is uncontested that no progress will be possible without strong health systems, and that strong health systems require adequate, well-distributed, appropriately trained, motivated and well supported and managed human resources (HR) [1-3]. Yet health systems in many low-income countries remain fragile, the human resource situations precarious, and the record of human resource planning and management of resources and capacity in Ministries of Health very uneven at best.

Causes of the continuing crisis include multiple contextual factors: a growing, and increasingly complex, disease burden; high levels of brain drain; demotivating working environments; rapid population growth; economic deprivation; poor health infrastructure; and civil and political unrest [3-7].

These factors are accompanied by a lack of multi-faceted and comprehensive HR strategies in countries. While the focus on HR leadership in the early 2000s has led to the development of national plans and policies for HRH in many countries, there has been a poor record of implementing these [3,8,9]. According to a study in 2009, 78% of the 57 countries, which are experiencing acute shortages of HRH, had HR policies and plans, and only a little over half (55%) of these countries had put these guidelines into practice [8].

Underlying HRH shortages and weak leadership capacity for HRH is another crisis: the poor capacity of most academic institutions in Africa. The 2005 Commission for Africa Report emphasized the dire state of African universities [10], many of which are ill equipped to train the next generation of health workers and leaders required in these countries.

Public health training is no exception: Ijsselmuiden et al. have reported [11] the dramatic inadequacy of graduate public health training in Africa. At the time of their report, in 2007, 29 countries had no advanced public health training programme locally, and 11 countries had only one programme/institution. Furthermore, in 2005, there were only 854 public health academics on the continent, only 493 of whom were full-time staff. Juxtaposing this with the capacity of countries in the North highlights a massive disparity: the author of Afrihealth quotes a colleague who stated that *"*the total academic public health workforce in Africa could fit into the department of epidemiology at Johns Hopkins*"* [11].

A brain drain of academic staff into well-paying nongovernmental organizations (NGOs) and ‘moonlighting’ through extensive consultancy work further aggravate the situation, taking time away from academic work and resulting in competing priorities and interests and misuse of resources [12-15].

Hence, public health training institutions face the dual challenge of mitigating the HR capacity deficits of their country while simultaneously battling the limits and continuing depletion of their own capacity [16].

In this paper, we report on one initiative aimed at addressing this dual capacity challenge through the development and implementation of a joint MPH programme with a focus on health workforce development. Four academic institutions from East and Southern Africa have begun to pool and collectively strengthen resources and build a joint teaching platform.

While capacity development is a long term and complex endeavour, some early lessons are worth sharing. In this case study we report on the history and rationale of the project and discuss achievements and challenges of building institutional and individual capacity to develop and deliver new forms of training for health workforce development.

## Methods

This paper presents data obtained through interviews and group discussions with the 18 trainees enrolled in the Masters in Public Health (MPH) programme at the University of the Western Cape as well as representatives of partner institutions. The paper draws on data from bi-annual anonymous surveys with students when they attended classes in Cape Town, focusing on their experience and perceptions of the training, academic support, and challenges encountered. The paper also draws on information from direct and participant observations of project meetings as well as reviews of publications and project documents, including evaluations, project proposals, agreements, progress reports, memos, minutes, and internal records. Questions guiding data collection were constructed by the project team as part of an on-going monitoring and evaluation activity and a doctoral research project, which are integral to the overall programme. Data obtained from the multiple sources were analysed using thematic analysis.

Ethical clearance to undertake the study was secured from the University of the Western Cape.

## Case description

### The consortium’s origin and configuration

In 2008, the World Health Organization (WHO) initiated and funded a consortium of academic institutions from four countries in East and Southern Africa to implement a co-operative intervention "to develop a sustainable masters-level educational programme with a focus on Health Workforce Development" [17]. It considered this part of the broader effort to "contribute directly to the rapid response that is required in order to address the critical [health workforce] situation", and "generate leaders who will spearhead the production and management of the health workforce for years to come" in sub-Saharan Africa.

Departing from conventional North–South cooperation, and drawing inspiration from Latin American and particularly Brazilian initiatives to strengthen public health and HRH capacity [18-20], the consortium is intentionally all-African, expressing the desire that institutions in the South should engage "in collaborative learning models to share innovative, adaptable and cost-efficient solutions to address their development challenges" [21]. It came about as a result of past institutional and faculty collaboration and the requirement for the educational programme to be delivered in English, French and Portuguese. The consortium, therefore, includes institutions from Anglophone, Lusophone, and Francophone Africa, namely:

• School of Public Health, University of the Western Cape (UWC), South Africa

• School of Public Health, Addis Ababa University (AAU), Ethiopia

• Department of Community Health, University of Eduardo Mondlane (UEM), Mozambique

• School of Public Health, National University of Rwanda (NUR).

All partner institutions have long histories in public health research and training in their respective countries (see Table 1). Despite their longevity and the significant contributions they are making to tackling health problems in their countries, they all have a very small staff complement that focuses on health systems development and HRH, in common with most academic institutions in Africa [22,23]. Furthermore, they all display the fragility of academic institutions discussed in the introduction, e.g. difficulties in attracting and retaining young academics.

**Table 1:** MPH focusing on health workforce development – profile of partner institutions

**Collaborating institution**	**Year established**	**Mode of postgraduate teaching**	**Language**	**Permanent staff complement**	**No of MPH, PhD students 2012**
School of Public Health, University of the Western Cape (UWC), South Africa	1993, MPH in 1994	Distance learning, short face-to-face courses	Anglophone	11	284 MPH 44 PhD
Department of Community Health, University of Eduardo Mondlane (UEM), Mozambique	1962 MPH in 2001	Face-to-face	Lusophone	12	37
School of Public Health, National University of Rwanda (NUR)	2001	Face-to-face	Francophone	18	176
School of Public Health, Addis Ababa University (AAU), Ethiopia	1964- community health department MPH initiated in 1984	Face-to-face, resource based	Anglophone	25	110 MPH 10 PhD

The consortium developed and implemented a ‘Masters Degree programme with a focus on health workforce development’ geared towards meeting the following two interrelated specific objectives:

• To strengthen the leadership capacity of Health ministries to develop expertise in HRH planning and management.

• To strengthen faculty capacity to teach about health workforce development through sharing and developing teaching resources, collaborative teaching and supervision, and developing an African platform for teaching policy, planning and the development of human resources for health.

**Figure 1:**
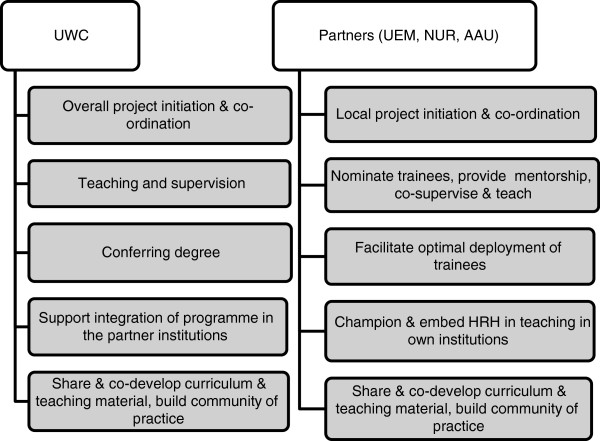
Role of partner institutions in the implementation of the programme.

The initiative was configured to facilitate sharing of experience and resources, and recognized the relative strength and assets of partner institutions in the area of HRH specifically and public health education in general. It built on the experience and practices of the School of Public Health (SoPH) at the University of the Western Cape, which has a long history of postgraduate training and distance learning on the continent [24,25] and has been serving as a WHO Collaborating Centre for Training and Research on Human Resources for Health since the early 2000s. The development of the new MPH programme was, thus, directly rooted in the development of postgraduate education at the school over the past two decades. The roles of the four partner institutions are detailed in Figure 1.

### Building on existing foundations

UWC started offering an MPH in 1994 as a structured part-time programme, and since 2000 as a distance learning programme, in response to the growing demand and public health challenges as identified by Ijsselmuident et al. [11]. From the start it was oriented towards meeting the needs of health professionals working in a context of growing decentralization and an increased focus on a primary health care (PHC) approach [24] - consistent with many African countries where the role of managing and coordinating the health system has continuously devolved from national and regional levels to the districts.

But "with long histories of centralized governance and underdevelopment of formal local governing" [26], capacity needs are not only great, but also complex and multi-faceted, having to address individual, organizational and systems capacity, as well as anything from technical skills to established mindsets and organizational ‘cultures’ [27].

Hence, the emphasis on improving capacity for HRH leadership and governance which are central to this initiative, have been the hallmark of UWC SoPH’s many years of engagement:

It is our own experience that public health education is most effective when it is problem-orientated, worksite-based and preferably organised around real challenges facing learners in their work situation….Success and sustainability are further enhanced if care is taken to root capacity development skills at local level. This may be facilitated by starting the training process with managers and supervisors who then act as change agents and are able to motivate others [25].

**Figure 2:**
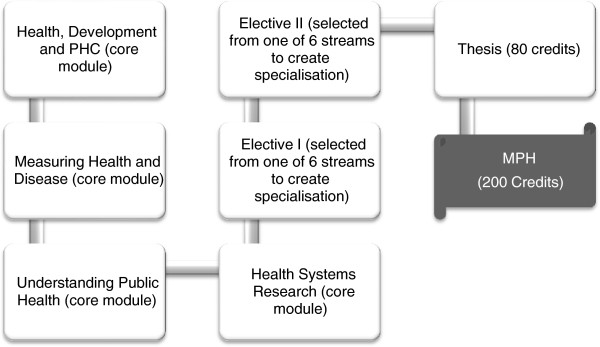
UWC Masters in Public Health (6 × 20 credit modules + 80 credit thesis).

**Figure 3:**
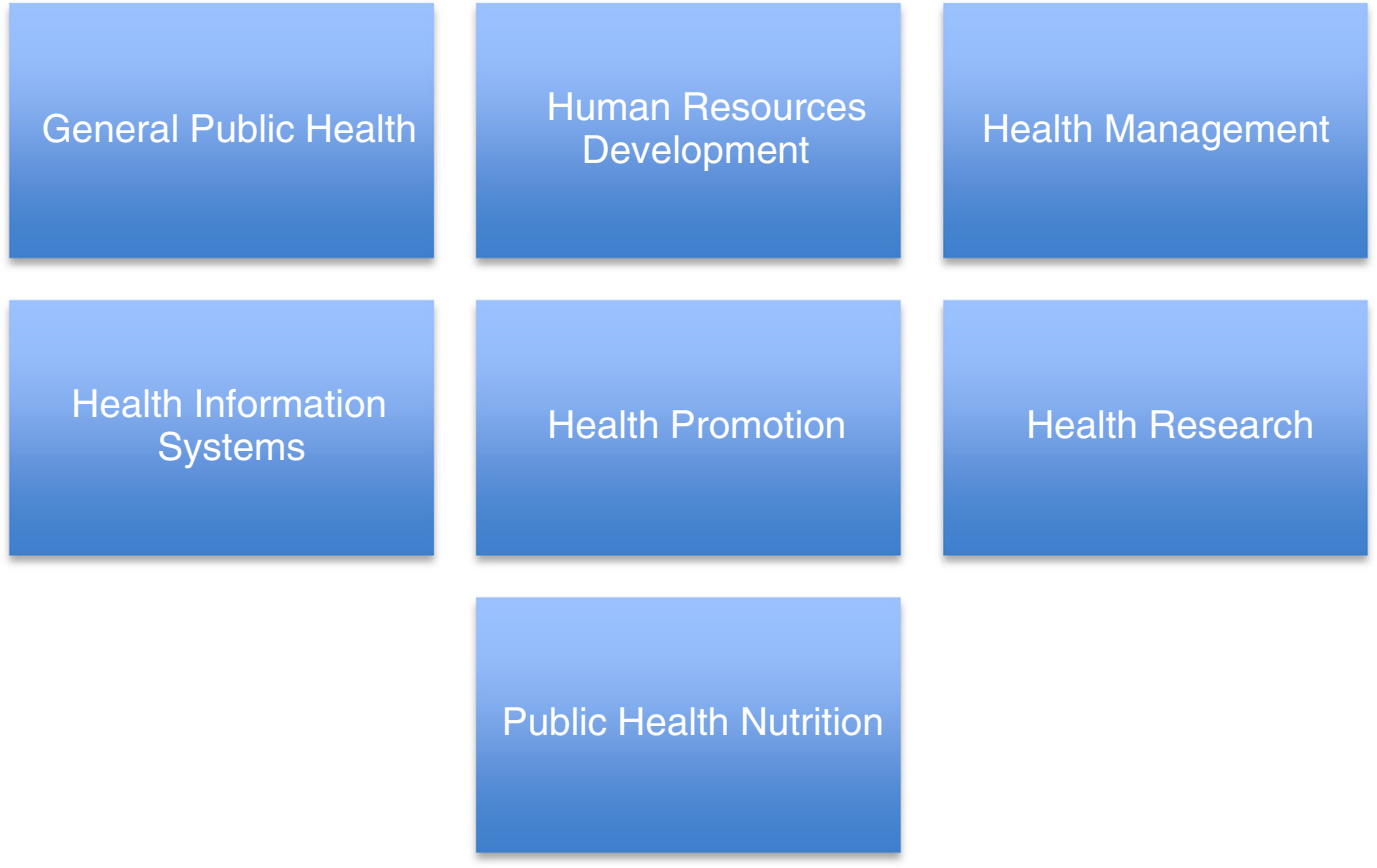
Streams/Specializations in UWC MPH Programme.

The UWC MPH programme is structured to consist of a series of core modules, to be followed by several electives in one of six specializations or streams (see Figures 2 and 3 below^a^). The programme is delivered in mixed and open mode, with most modules available as distance courses, which consist of working through learning guides, submitting assignments, participating in Google discussion groups and e-mail engagements with lecturers. In addition, many courses also have an optional face-to-face component during the SOPH’s Summer and Winter Schools. Thesis supervision takes place by e-mail and participation in a face-to-face thesis week when students develop their research question and objectives and deepen their research skills.

The School’s learning approach has always been geared towards working practitioners [25], and the move to an open and distance mode in the early 2000s has provided access to many practitioners who otherwise would not be able to pursue postgraduate studies. Gains in access, however, do not come without cost: isolation of learners, lack of support, and grappling with how to teach and supervise application of skills have remained challenges for this programme [24], as they are for distance learning programmes internationally [28-30].

With UWC’s experiences and programme structures providing the foundations for collaboratively building regional institutional capacity, this project presented an opportunity to address some of the enduring challenges of offering a professional training programme through open learning and to experiment with new forms of student support. In particular, the project aimed to introduce local mentoring support, to work with cohorts of learners, and to ensure internet connectivity of all students, while building joint teaching capacity.

### Student enrolment and support

The project intended to enrol small teams of students from each country’s ministry of health to facilitate peer learning and reduce isolation, a central success factor in distance learning [28,31] and to create a small "critical mass" in their organizations to initiate change and innovation. An unanticipated challenge, however, was the intervention of ministries of health in the selection of students. In all three countries ministries insisted that routine public service protocols be followed in advertising the study opportunity and selecting students. While this improved awareness of the programme, it undermined a central aim of the project: students studied as individuals, rather than in teams, and they could not easily apply and practice newly developed skills in their work places.

Altogether eighteen students (six each from Ethiopia, Mozambique and Rwanda) were enrolled in two consecutive intakes of the UWC MPH in 2010 and 2011. Thirteen students occupied high-level positions in ministries of health or their substructures while five were from training and research institutions. They all had medical or social science backgrounds. Students completed their course work within the structure of the UWC MPH programme (see above), taking three HRH electives and preparing a mini-thesis with an explicit focus on a HRH topic in their country (see Table 2).

**Table 2:** Mini-thesis titles of graduates of the MPH on health workforce development

Mozambique	The perceptions and experiences of medical technicians on the decentralization of the ART programme in Mozambique
	Brain drain of medical doctors in public health sector in Maputo city, Mozambique
	Perceptions and experiences of motivation among nurses in Maputo Central Hospital
	Impact of GHI in Human Resources for Health in terms of sustainability and performance
Rwanda	Factors that influence intent to stay among health workers in Kabaya, Rwanda
	Effectiveness and challenges of ART scale up through task shifting in art services delivery: case of primary health centres under supervision of Kibagabaga District Hospital
	Factors affecting performance of maternal health care providers in Kibagabaga District Hospital, Rwanda
	Job satisfaction of Health workers in Kigali University Teaching Hospital
	Predictors of burnout among nurses in paediatric and maternity wards of district hospitals of Kigali city in Rwanda
Ethiopia	Utilization of the Health Extension Programme services in Akaki District, Ethiopia
	Determinants of nurses’ motivation in Butajira Zone Hospital, Ethiopia
	Challenges that face health extension workers in their work in Bahirdar District, Ethiopia
	Assessment of job satisfaction among physicians working in hospitals, Addis Ababa, Ethiopia
	Assessment of the effect of place of selection on performance of health posts and turnover of health extension workers in Jimma Zone, Ethiopia
	Factors affecting career intentions of medical students in two medical schools in Addis Ababa, Ethiopia

After the first intake and a review of early lessons of implementation, the experiences with student selection, and challenges to find local capacity to champion and embed the project, an important shift was made. Recognizing the very limited capacity of academic institutions in offering public health training in the field of HRH, it was decided that a second intake would focus on training young academics in teaching, curriculum development and academic leadership in HRH; thus, five students were selected from training and research institutions. In this way, building the capacity of academic institutions later gained prominence over the building of capacity in Ministries of Health.

The other … aspect of sustainability, which came more to the fore after the inception of the programme is our recognition that we need to build capacity in training institutions in those countries in order that, after the end of this project, they could, if they wish, replicate what we do - not necessarily exactly but at least be able to offer some components of the MPH which we have developed (Interview with senior staff member 1, UWC SOPH, 2012)*.*

In addition to access to UWCs’ MPH programme, students received extensive additional support, including funding for travel to attend face-to-face sessions, for laptops and internet support, and local mentorship support (discussed below).

We conducted regular anonymous surveys with students when they attended classes in Cape Town to gauge satisfaction, but more importantly as a learning and continuous improvement measure. They explored students’ satisfaction about the face-to-face and distance training, relevance of knowledge acquired to their own work/context, feedback on academic support and particularly mentorship, as well as challenges encountered. We found that students were particularly appreciative of the relevance and applicability of the curriculum, filling a distinct skills gap in their career trajectory into management or academic positions. A trainee recounted the relevance of the training to his work as follows,

… Most people in my opinion get their managements skills either by experience …. You know with working in the bureaucracy for some time. But me with the readings that I have had, … I think it has given me a lot of insight and a lot of help in terms of how to manage … not only insights to the problems, but also insights to the solutions as well.

We [members of an intersectoral task team led by MOH/HRH unit] are planning to conduct supportive supervision to … medical schools … So … we have put a detail plan to do this supportive supervision. And readings [from one of the HRH modules] … have been very helpful for me because I was the one who was planning the detail strategic planning … It helped me. The readings helped a lot in understanding what is expected from me and what possible challenges could come…. And what possible solutions should I look for (Interview with trainee, MoH-Ethiopia, 2012)*.*

However, students also identified numerous challenges during the programme, including lack of time to focus on their studies due to employers’ reluctance to allow time off work, lack of mentor support, language barriers (as the MPH is offered in English), and weak academic writing skills. Many of these challenges are common to mature and working students [32,33]. While some of these could be addressed immediately, such as substantially increasing language and writing support, others require long-term planning and are part of an ongoing endeavour to improve educational delivery for professional continuing education.

To date, fifteen students have graduated with this MPH and there is evidence they are applying what they learnt in their work context - a case in point being the initiation and development of a national HRH development unit in the MoH in Ethiopia under the leadership of graduates of this programme.

### Sharing and local adaptation of curriculum and teaching materials

Over the years, the UWC SoPH has developed detailed learning guides and readers for the majority of the more than 20 modules which form its distance teaching programme. The teaching materials constitute module guides, which use scaffolding and a ‘guided didactic conversation’ approach [24,30,34,35]. The guides are complemented by a reader, which is a compilation of important course readings. The module guides are characterized by questions that guide students through the learning process, and by tasks that require students to "integrate and apply new concepts, models, strategies and approaches to common practical problems frequently encountered by managers and practitioners in the health services … which facilitates the immediate application of theoretical concepts and models to their situations in the work arena" [24]. These materials undergo revisions on average every three years, and learners’ experiences and needs, as well as the regular research the institution conducts, provide the all-important feedback in this regard.

The research we do in this field, and also all the research we do at the School, is also very, very applied because we are working closely with facilities, with sub-district managers, with district managers. We have a pretty good feel what the issues are, and I think that is probably what students appreciate when they talk about relevance; the cases we use, the questions we raise, resonate with the questions and the challenges they face in their work. And obviously we also then continuously learn from students writing their thesis, the inputs they give in courses and so on; constantly rework and develop our own thinking. … I think generally our courses try to be very close to practice, [and] we use as our learning principle the respect and understanding that people are experienced professionals (Interview with senior staff member 2, UWC SOPH, 2013)*.*

As part of this project, some of the general MPH and specialist HRH modules were adapted for the other local national contexts for use in the partner countries, while others were translated from English to Portuguese. Presently, four modules are under development and/or revision in English, while seven have been translated from English into Portuguese and are being used in a distance learning MPH programme on Health Workforce Development launched in June 2013 by the Department of Community Health, Eduardo Mondlane University in Mozambique.

The programme’s initial plan to make available teaching material in French was cancelled as Rwanda joined the Commonwealth [36] and adopted English as the official language of instruction in 2008 in a bid to harmonize its curriculum with its English-speaking East African Community [37]. Instead, WHO initiated a similar HRH curriculum and training initiative focusing on Francophone Africa, which is led by the University of Geneva.

All materials, as well as other modules of UWC’s MPH programme are presently being made available to a wider audience as ‘share-alike’ free courseware to encourage their use by other academic institutions in the target countries and beyond that seek to strengthen their programmes^b^.

To support partners as they began to explore delivery modes for their institutions, UWC organized a workshop on open and distance learning (ODL) focusing on course and curriculum development generally and the pedagogical, administrative and financial aspects of distance learning more specifically. Participants were ‘champions’ in their own institutions, identified to lead the sensitization of colleagues to distance learning and its relevance, to advocate for its adoption in the institution and to play a part in its implementation. In the course of the workshops they had the opportunity to design strategies for materials development and support that suit their contexts and institutions’ needs. The importance and the complexity of building curriculum and teaching expertise constitute one of the key lessons from the programme, which will be discussed further below.

Despite the numerous benefits, there were multiple challenges in the process of developing and implementing distance learning education, ranging from policy and organizational challenges to issues related to financial and student support. UWC’s experience is instructive in this regard:

There have been the well-documented organisational challenges to the delivery of a distance learning programme …in a university originally structured around contact and residential training. … It is often difficult to align administrative systems, and … teaching and learning activities *…*[24]*.*

Representatives of partner institutions who attended the ODL workshop expressed similar sentiments. They foresaw a range of challenges in implementing distance education in their respective institutions including the policy environment, a lack of capacity and reluctance among staff to embrace distance learning, a lack of capacity for materials development and student support, and issues of funding and financial management. One example of the range of challenges to be expected is the Ethiopian Federal Education Ministry’s decision in August 2010 to ban distance learning offered by both public and private universities due to "quality concerns" [38]. While it lifted this ban again in October of the same year, the intervention created uncertainty in planning a distance learning programme. Developments such as these are indicative of the unpredictable and dynamic nature of the policy environment and the constant tension between education coverage and quality.

However, such challenges run alongside steady progress: one institution (UEM) has already started implementing distance learning, supplemented by face-to-face contact sessions, with an initial intake of 27 students. Two institutions (NUR and AAU) are in the process of developing alternative ‘local fits’, such as HRH certificate programmes, as a start.

### The centrality of information and communication technology to access and communication

Academic institutions are under increasing pressure to adapt to a range of global transformations, including decline in the global economy accompanied by rises in the cost of education, growing global integration, constant advances in technology, and the need for greater flexibility in educational delivery [39]. Despite the growing use of information and communication technologies (ICTs) as a platform for teaching and learning, the lack of ICT infrastructure in African countries and limited e-skills of learners continues to undermine the benefits of these technologies. This poses a dilemma for academic institutions wanting to improve access to their programmes through distance and e-learning as dispersed and remote students continue to have the least access to ICT infrastructure and skills.

The gap in access to, and quality of, internet connectivity in Africa was highlighted in a recent study about the state of HRH units in MOHs at a national level, which found that individual staff has access to a personal computer in only 27% of the countries surveyed. While units in 84% of the countries have access to the internet, individuals have reliable access in only half of the countries [8]. It is safe to conclude that the situation of such units at sub-national levels is far worse.

Poor connectivity and low bandwidth have also been main challenges in this programme, impeding regular use of even routine e-mail exchanges and internet-based calls. While students in the programme have varying levels of e-skills, the fact remains that access is a challenge for many and that those based outside capital cities fare worse in this regard. The project’s intentions to alleviate isolation and nurture communication through the enrolment of small groups of students in geographical and institutional proximity with each other was undone by countries’ needs to employ standard public service regulations to advertise the programme and enrol students. Instead, it opted for well-tested avenues of communication with students, combining the face-to-face contact sessions and the use of digital and print media with e-mails and phone calls [35] and an effort to establish a mentorship programme.

This blended approach has mitigated some of the impact of the lack of ICT infrastructure and expertise, which has been particularly hard for students based outside the capital cities, as is the case in many such programmes [40]. But balancing improvements in access with appropriate delivery remains one of our key challenges.

### Mentorships

Since "not all competencies can be developed without some interaction with trainers or peers", and aware of the "loneliness" of studying at a distance and the challenges of translating theory into practice [24,25,41], UWC SoPH had for several years explored mechanisms to support students in their studies and in the application of new knowledge and skills in their professional contexts. Previous attempts to recruit alumni as volunteer mentors had not succeeded as mentor commitments invariably clashed with work commitments of these busy managers. This programme provided the opportunity to revive the mentorship idea by providing funding for training and stipends of two mentors, who were chosen by the institution in each country to support local students. All were senior professionals, with three being based at academic institutions, one in the ministry of health and two being independent consultants linked to partner institutions.

These local mentors were expected to play a central role in the programme: to accompany students from the time they enrolled by tracking their progress, assisting them with study material, helping with assignments and theses, and discussing with them how to apply newly acquired knowledge and skills in their work places. Each mentor was expected to support three students on average, and each student was expected to receive an hour’s support every week from the mentor. Mentors were, thus, expected to spend around three hours per week with their students. Each partner institution received a small fund for mentor stipends, meant to provide a modest salary top-up rather than a salary. However, the disbursement of funds was left to partner institutions and handled differently in each case. All mentors received one week of orientation and training at the University of the Western Cape.

In practice, the recruitment of mentors and retention of their commitment and support proved one of the greatest challenges of the project. Difficulties experienced by mentors included establishing regular contact, organizing meetings and providing support to students, especially to those located outside the capital city, and those with little or no internet connectivity.

From the students’ point of view, one of the challenges was failure of the mentors to provide adequate and timely guidance on all six modules, as they were HRH specialists rather than generic public health teachers. The partner institutions tried to deal with this challenge by involving other staff in the institution or organizing special sessions for students.

Most importantly, it proved difficult to find mentors with both the expertise and the time to dedicate to students. Most mentors who were appointed were extremely busy academics or professionals with multiple competing commitments and priorities. Against this background, and despite training and the availability of stipends, active mentorship was mostly a low priority, bringing into stark relief the conundrum of having to build capacity with very limited capacity [12,42].

## Discussion

The implementation of this collaborative initiative has been complex, straddling multiple and changing contexts, actors and agendas. Some of these are common to postgraduate programmes enrolling working students from different countries, such as weak language and academic literacy skills and lack of time for study, lack of capacity to provide sufficient support to students and the variable provision of reliable ICTs. Others are unique to this particular partnership programme, such as weak institutional capacity in the partner institutions to champion and embed the programme; competing and changing institutional priorities; and the need to navigate institutional and country contexts, such as new policies and decision-making processes.

### Building capacity of academic institutions

The initiative set out to build capacity in, and a joint teaching platform for, training in health workforce development by collaboratively training small cohorts of learners located in MoHs, while simultaneously developing capacity in academic institutions. This approach underestimated the challenge of building sustainable institutional capacity. While all partners had in-principle institutional support for the initiative, only a small number of staff found the time to dedicate to the project and to build wider institutional commitment to develop new forms of training through flexible delivery and open learning approaches. Furthermore, it became evident that expertise in curriculum development and innovative educational practices was a major capacity gap. Both insights led to a shift in the project’s focus from building capacity in MoHs, while also building capacity in academic institutions, to focusing predominantly on the latter. The project re-allocated funding to pay for full-time champions in each partner institution, with the brief to guide curriculum and course development and build staff capacity. Furthermore, attention and resources were focused specifically on building capacity in curriculum and materials development, as well as administrative expertise for the implementation of open and distance learning programmes in public health and specifically health workforce development. Relevant short courses and manuals are presently being developed as the project moves into its final phase of implementation.

### A key challenge: mentorship support

Support for working adults studying at a distance has been one of the central and most challenging goals and ambitions of UWC’s MPH programme generally, and this is reflected also in the MPH in Health Workforce Development. Given the shallow pool of senior academics and practitioners on the continent who are both able and available to provide supervision, mentoring and coaching support, building robust networks of mentors remains a significant constraint to capacity building generally and has proved to be the case here. Incentives, such as training and salary top-ups, proved insufficient to attract and retain mentorship support, indicating a need to rethink and improve both. However, it also suggests a lack of understanding and appreciation of the importance of curricular and learning processes in open and distance learning [28,29,31,34]. This gap is now being addressed through the development of dedicated courses and concerted efforts to build and nurture an alumni network in the region, which can become a skilled pool of mentors in the future.

All efforts to build individual and institutional capacity continue to take place in the context of severe resource constraints and competition for scarce human resources. Academic institutions constantly struggle to secure funding [43] to support their research, teaching endeavours, or sustain programmes of this type, while simultaneously battling to build the next generations of researchers and teachers [15]. The systematic strengthening of the academy is rarely a priority. As the 2010 Commission for Africa testified, there is not only a lack of national investment in African universities, but external funding also continues to be extremely low [44].

The greatest challenge, however, lies in increasing access (numbers of students) and ensuring student support and success without jeopardizing quality through premature and unsupported massification of programmes, as has become a recent tendency with some online training programmes [39]. Building capacity is an iterative and painstaking process and long-term endeavour, requiring both sustained institutional commitments and substantial resources. It is the explicit intention and ambition of this partnership to establish a platform for this endeavour beyond the lifetime of the project. The aim is to develop mechanisms that embed capacities to plan, develop and manage HRH and to train for such capacity in both ministries of health and academic institutions.

## Conclusions

A solution to the health systems crisis in many African countries requires multiple responses. Key among these is strong leadership in planning for and managing human resources and dramatically improved capacity of academic institutions to train health workers and health systems leaders.

This situation poses a challenge, which four African universities, with support from WHO, are trying to address in a partnership aimed at strengthening collective capacity to train leaders in HRH.

While there are significant inherent challenges, the programme has potential for providing real opportunities for building the field and community of practice, and strengthening the staff and organizational capacity of participant institutions. The aim is that it will ultimately have impact in the region.

It is too early to judge the likely sustainability of this initiative. Apart from availability of funding, this will depend on the intensity and nature of cooperation among the institutions, the commitment of the leadership, and the degree to which key stakeholders and staff are enrolled in nurturing and building the partnership [16]. It has become apparent that more time is required to embed aspects of the programme in some of the partner institutions, due to complex institutional processes and stakeholder relations, a difficult policy environment, and weak institutional capacity to champion it.

The initial decision to conceptualize the initiative as an African endeavour has proven to be both a challenge and an opportunity. It is ultimately crucial to developing sustainable capacity in the region. The nature of cooperation and the focus on institutional capacity has been one of the project’s greatest achievements. This cooperation was configured to be led by southern partners, and has involved the transfer and exchange of knowledge and experience among institutions facing similar problems. Such initiatives remain in the minority [45], but are on the ascendency [21].

This paper emphasizes the need for long-term strategies and engagement, more investment and attention to developing the capacity of academic institutions, the need to invest specifically in educational/teaching expertise for innovative approaches to teaching and capacity development more broadly, and the importance of increasing access and support for students who are working adults in public health institutions throughout Africa. In 2007 Paulo Buss reported on Brazil’s success in strengthening public health education and HRH leadership through proliferating schools of public health in the country [18]. Africa should aim for similar success stories.

## Endnotes

^a^The course structure has been slightly changed since then, with students now taking altogether eight modules which each are shorter and carry fewer (fifteen) credits (four core modules, two research modules, and two elective modules). The thesis component remains the same.

^b^These materials already are, or will soon be, available on the website of the UWC School of Public Health ( http://www.uwc.ac.za/Faculties/CHS/soph), the Open Education Resources website ( http://www.oercommons.org/) and the website of the Consortium for Health Policy and Systems Analysis in Africa ( http://www.hpsa-africa.org/).

## Abbreviations

AAU: Addis Ababa University; ICT: information and communication technologies; HRH: human resources for health; MoH: Ministry of Health; NUR: National University of Rwanda; ODL: open and distance learning; SoPH: School of Public Health; UEM: Eduardo Mondlane University; UWC: University of the Western Cape; WHO: World Health Organization.

## Competing interests

The authors declare that they have no competing interests.

## Authors’ contributions

All authors jointly conceived of the article. WKA and UL had primary responsibility for the draft of the manuscript. WKA, DS and UL all contributed substantially to the intellectual content, writing and finalization of the manuscript. All authors read and approved the final manuscript.
